# Combining Stressors That Individually Impede Long-Term Memory Blocks All Memory Processes

**DOI:** 10.1371/journal.pone.0079561

**Published:** 2013-11-06

**Authors:** Sarah Dalesman, Hiroshi Sunada, Morgan Lee Teskey, Ken Lukowiak

**Affiliations:** Hotchkiss Brain Institute, Department of Physiology and Pharmacology, University of Calgary, Calgary, Canada; Florida State University, United States of America

## Abstract

The effects of stress on memory are typically assessed individually; however, in reality different stressors are often experienced simultaneously. Here we determined the effect that two environmentally relevant stressors, crowding and low calcium availability, have on memory and neural activity following operant conditioning of aerial respiration in the pond snail, *Lymnaea stagnalis*. We measured aerial breathing behaviour and activity of a neuron necessary for memory formation, right pedal dorsal 1 (RPeD1), in the central pattern generator (CPG) that drives aerial respiration in untrained animals, and assessed how these traits changed following training. In naïve animals both crowding and combined stressors significantly depressed burst activity in RPeD1 which correlated with a depression in aerial breathing behaviour, whereas low calcium availability had no effect on RPeD1 activity. Following training, changes in burst activity in RPeD1 correlated with behavioural changes, decreasing relative to their naïve state at 3 h and 24 h in control conditions when both intermediate-term memory (ITM: 3 h) and long-term memory (LTM: 24 h) are formed, at 3 h but not 24 h when exposed to individual stressors when only ITM is formed, and did not change in combined stressors (i.e. when no memory is formed). Additionally, we also found that *Lymnaea* formed short-term memory (STM: 10 min) in the presence of individual stressors or under control conditions, but failed to do so in the presence of combined stressors. Our data demonstrate that by combining stressors that individually block LTM only we can block all memory processes. Therefore the effects of two stressors with similar individual affects on memory phenotype may be additive when experienced in combination.

## Introduction

Memories shape the way in which an individual interacts with its environment. Stress experienced before, during or following a period of learning can enhance or block memory formation depending on the nature of the stress [1], [2]. Different stressors can have similar effects on memory phenotype; however, the way in which each of the stressors affects the central nervous system (CNS) may differ [3], [4], and therefore we cannot always make *a priori* predictions about how combining stressors will alter memory phenotype based on their individual effects [5]. Typically, in the laboratory the effects of individual stressors are determined in isolation, but in ‘real life’ multiple forms of stress may be experienced simultaneously. Therefore, we addressed whether the effect on memory following exposure to multiple forms of stress with identical individual effects on memory phenotype are additive when combined.

We use operant conditioning of aerial respiration in *Lymnaea* to assess the effects of various stressors on memory formation at both the behavioural and individual neuron level (e.g. right pedal dorsal 1; RPeD1) [1], [6]. *Lymnaea* is a pulmonate snail. In high oxygen conditions it breathes cutaneously; however, in hypoxic conditions it switches to aerial respiration using a basic lung opened via the pneumostome [7]. RPeD1 is part of the central pattern generator (CPG) that drives aerial respiratory behaviour [8]. Changes in RPeD1 burst activity have been demonstrated to be necessary in altering aerial respiratory behaviour following training [9]–[11]. Therefore we can directly measure memory formation following operant conditioning of aerial respiration at both behavioural and neuronal levels in *Lymnaea*.

We have previously shown that memory enhancing stressors, predator kairomones and KCl, which are modulated via the same sensory system, have similar effects on the activity of RPeD1 in naïve *Lymnaea* [12]. Here, we assess the effects of two alternative stressors, crowding and low calcium availability. These stressors may be experienced in combination by *Lymnaea* in the natural environment, when high population density coincides with low calcium levels in natural water systems [13]–[15]. Individually they block long-term memory (LTM) whilst allowing intermediate-term memory (ITM) formation [11], [16], [17], but are modulated via different sensory systems [18]. Additionally, their effects on alternate ‘smart’ strains of *Lymnaea*, considered strains that are able to form long-term memory (LTM) following a training regime that only produces intermediate-term memory (ITM) in the Dutch strain used here, differ. In these ‘smart’ strains low calcium availability does not block LTM whereas crowding does [19]. We predicted based on their effect on ‘smart’ strains that crowding and low calcium availability may differ in how they alter activity in RPeD1 despite producing an identical phenotypic effect on memory formation in the Dutch strain. The effects of combined stressors may not be easily predicted from their individual properties [5], so we also assessed whether combining these stressors would produce additive results, blocking earlier memory processes.

## Materials and Methods

Adult *Lymnaea stagnalis* (spire height 25±1 mm) from a strain originating from animals collected in a polder near Utrecht, Netherlands in the 1950s (the Dutch laboratory strain) were reared under standard conditions in the Biological Sciences building at the University of Calgary [20]. Snails were transferred to the laboratory a minimum of 1 week prior to the start of experiments and maintained in oxygenated artificial pond water (APW) made from deionised water with the addition of 0.26 g/l Instant Ocean® (Aquarium Systems Inc., Mentor, OH, USA) and calcium sulphate dehydrate to provide a calcium concentration of 80 mg/l [21]. Snails were kept at room temperature (20±1°C) at a stocking density of 1 snail per litre (uncrowded conditions) on a 16:8 light:dark schedule and fed romaine lettuce *ad libitum*.

### Stress exposure

We used two stressors, low calcium availability and crowding, both of which block LTM but allow ITM formation when experienced individually [11], [16]–[19]. Stress exposure was used both individually as in previous work, and also in combination, to assess their effects on memory of operant conditioning to reduce aerial respiration in *Lymnaea*. In control conditions snails were maintained in our standard calcium artificial pond water in uncrowded conditions throughout. To expose snails to low calcium (20 mg/l) we transferred them into low calcium artificial pond water 1 week prior to training, containing 0.26 g/l Instant Ocean and with calcium sulphate added to provide 20 mg/l [Ca^2+^] [21]. Snails were also trained and tested in low calcium conditions. Crowding stress was produced by placing 20 individuals into 100 ml of pond water in a 1 l glass beaker for 1 h immediately prior to the first training session [16]. When stressors were combined, snails were maintained for 1 week in low calcium conditions, and then crowded for 1 h immediately prior to the first training session in low calcium water. To assess the electrophysiological response in RPeD1 to stress in the absence of training in naïve animals (see below), animals were stressed as above then anesthetised to allow RPeD1 activity to be recorded 3 h following stress exposure.

### Breathing behaviour

*Lymnaea* are pulmonate snails. In eumoxic conditions they primarily breathe cutaneously, absorbing oxygen across their skin; however, in hypoxic conditions they move to the water’s surface and breathe using a lung opened to the air via the pneumostome. Low calcium conditions and crowding have both been found to depress aerial breathing behaviour in hypoxia in the absence of physical stimuli when each stressor is experienced in isolation [16], [21]. However, we also wanted to assess whether combined stressors would have a similar affect on aerial respiration. Breathing behaviour was assessed in two groups, one maintained in control conditions throughout, and the other exposed to combined stressors (maintained in low calcium for 1 week and crowded for 1 h). *Lymnaea* breathing behaviour was assessed under control conditions initially by placing them into hypoxia for a 10 min acclimation period, then measuring the total breathing time (TBT) over 30 min (pre-Obs) [21]. The snails were then divided randomly into 2 groups, one of which was exposed to control conditions for 1 week, and one exposed to low calcium followed by 1 h crowding. We then assessed post-exposure breathing behaviour (post-Obs) at 10 min, 3 h and 24 h following stress exposure (equivalent to time periods when memory is measured following training). Breathing behaviour at 3 h and 24 h is also directly comparable to the time periods when we assessed activity levels in RPeD1 (see below).

### Training protocol

Operant conditioning of aerial respiration was carried out in the same way for all experiments [7]. 500 ml of artificial pond water, with either 80 mg/l or 20 mg/l [Ca^2+^] added depending on treatment protocol, was placed in a 1 l glass beaker. N_2_ was then vigorously bubbled through the water for 20 min to make the water hypoxic (< 5% [O_2_]). N_2_ bubbling was reduced and continued at a low level to maintain hypoxic conditions without disturbing the animals. Snails were then introduced into the beaker and allowed to acclimate for 10 min before the start of training. Training was carried out for 30 min (TR1), whereby the snail receives a tactile stimulus (a poke) on the pneumostome each time it attempts to open it at the water’s surface. This poke is sufficient to cause the pneumostome to close, but does not cause the snail to withdraw into its shell. To test for short-term memory (STM) formation the snails were moved immediately following TR1 into a second beaker, allowed to acclimate for 10 min, and then received the 0.5 h memory test (MT) immediately following this acclimation period. Following STM training, memory was considered present if pneumostome openings are significantly lower during MT than during TR1. To test for intermediate-term memory (ITM) and long-term memory (LTM) the snails were returned to their eumoxic (∼100% [O_2_]) aquaria for 1 h following the first training session (TR1), after which they received a further 30 min training session (TR2) identical to the first. To test for ITM or LTM we used an identical protocol to the first training session either 3 or 24 h respectively following TR2. The number of times the snail attempted to open its pneumostome during both the first (TR1) and second (TR2) training session and the test (MT) session were then compared to assess whether the snails had learnt and formed ITM or LTM. The snails are considered to have demonstrated learning if the number of attempted openings is significantly lower during TR2 than TR1. If the snail demonstrates ITM or LTM then the number of attempted pneumostome openings during the test session is both significantly lower than during TR1 and not significantly higher than during TR2.

### Yoked controls

To ensure that changes in breathing behaviour were due to the animals forming memory rather than a general depression in aerial respiration due to exposure to hypoxia or a physical stimulus we carried out yoked controls under control conditions. In this case the ‘yoked’ snail was paired with one that underwent normal training, and the yoked snail is then poked when the trained animal opened its pneumostome during both training sessions, i.e. pokes for the yoked snail are not contingent with pneumostome opening. In yoked controls for ITM and LTM as the trained animal received fewer pokes during the second training session, so did the yoked animal. Both trained and yoked individuals were then poked contingent to their own pneumostome opening during the memory test (MT).

### Electrophysiological activity in RPeD1

The necessity and sufficiency of right pedal dorsal 1 (RPeD1), a neuron in the central pattern generator that controls aerial respiration, has been demonstrated in driving aerial respiratory behaviour. Previous work has shown that the state of RPeD1 in naïve animals can indicate their ability to form LTM prior to training [12], [22], and that this state also changes following a training procedure that is sufficient to alter breathing behaviour [10], [23]–[26]. Therefore, we assessed RPeD1 parameters in naïve snails under control conditions, 3 h following exposure to each stress alone (low calcium or crowding) and following exposure to combined stressors. To assess RPeD1 activity in trained animals we carried out training (as above) in *vivo* under each of the four stress conditions, and then returned the animals to their aquaria. *Lymnaea* were then dissected following training so that the period during which RPeD1 activity was recorded occurred at the time when we would test for either ITM (3 h) or LTM (24 h) in intact animals. Data were not collected from RPeD1 at 10 min post-training (STM) as the time required to dissect the semi-intact preparation and allow for recovery is not short enough to enable recording within this time period. The parameters we measured were membrane resistance, excitability and bursting activity (a burst is required to initiate pneumostome opening). RPeD1 activity was recorded from semi-intact preparations, maintaining connectivity within the central nervous system (CNS) and between the CNS and a large proportion of the periphery including the pneumostome area.

The semi-intact preparations were dissected in a similar manner to that described previously [11], [23], [26], [27], except that the head/foot complex and buccal mass were removed. Preparations were pinned down in individual recording dishes in *Lymnaea* saline (51.3 mM NaCl, 1.7 mM KCl, 1.5 mM MgCl_2_, 4.0 mM CaCl_2_, and 10.0 mM HEPES, pH 8.0) with their dorsal sides uppermost. The central ring ganglia (CNS) were pinned to the dish directly. The outer sheath surrounding the CNS was removed using fine forceps. Standard intracellular recording techniques were used as described previously in *Lymnaea* semi-intact preparations [10], [11], [22], [26] using a saturated K_2_SO_4_ solution filled glass microelectrode with input resistance ranging from 15 to 50 MΩ. Voltage signals were amplified by a Neurodata IR283 amplifier (Cygnus technology, Inc, Delaware Water Gap, PA, USA) and displayed simultaneously on a Macintosh (Apple Computers, Cupertino, CA) PowerLab/4SP (ADInstruments Inc, Colorado Springs, CO, USA) and a storage oscilloscope (5113, Tektronix, Beaverton, OR, USA). Recordings were stored and analyzed via LabChart 7 software (ADInstruments Inc, Colorado Springs, CO, USA). Once RPeD1 was successfully impaled, the cells were given a 10 min stabilization period prior to recording. The electrode balance was measured at the beginning and end of each experiment and if the resistance had changed by more than 10% the trace was discarded.

Following stabilization, a 600 s trace was used to analyse bursts of action potential from RPeD1. Here a burst is defined as a period of sustained depolarization during which 2 to >20 action potentials are fired [11]. This definition has been derived from prior data collected on the properties of RPeD1, demonstrating bursts of action potentials that trigger pneumostome opening in semi-intact preparations of *Lymnaea*. Following trace recording, the excitability and input resistance of RPeD1 were recorded following previously described methods [11]. A measure of RPeD1 excitability was obtained by counting the number of action potentials elicited when the impaled cell was driven through a series of 10 depolarizing current steps, from 0.2 to 2.0 nA. Each step was 400 ms long, and the cell was allowed to recover for 300 ms between steps. Membrane input resistance (R_m_) was assessed by the slope of I-V relationships with injection of 10 hyperpolarizing current steps from –0.2 to –2.0 nA (step lengths are the same as above).

### Data analysis

All data were analysed using SPSS 17.0 (SPSS Inc., Chicago, IL, USA). Homogeneity of variance was confirmed prior to each test using Mauchly’s test for Sphericity prior to repeated measures ANOVA and Levene’s test prior to other analyses.

Data for total breathing time (TBT) 10 min post-stress were analysed separately from data for ITM and LTM to conform to other analyses on memory (see below). TBT during the initial observation (PreObs) vs. second observation (PostObs) was used as the within-subject factor and stress exposure (control vs. combined stressors) as the between-subject factor in rmANOVA. To assess whether stress altered breathing rates 3 h or 24 h post-exposure, TBT during PreObs vs. PostObs was used as the within-subject factor, with stress exposure (control vs. combined stress) and time at which PostObs was carried out (3 h vs. 24 h) as between-subject factors in rmANOVA. Post-hoc paired t-tests were used to assess whether TBT differed between the PreObs and PostObs where an overall significance was found.

Repeated measure ANOVAs (rmANOVA) were used to analyse the behavioural response to operant conditioning compared to yoked controls. STM was analysed separately from ITM and LTM as these animals only underwent a single training session, whereas ITM and LTM, which undergo identical training protocols, were assessed in a single analysis to directly compare the different memory durations. In assessment of yoked control data for STM, the response to training (TR vs. MT) was used as the within-subject factor, and training protocol (trained vs. yoked) was used as the between-subject factor. To assess ITM and LTM, the response to training was used as the within-subject factor (TR1 vs. TR2 vs. MT), and time at which memory was tested (3 h vs. 24 h) and training protocol (trained vs. yoked) as the between-subject factors.

In assessment of the response to training under different stress exposures at 10 min (STM), the response to training (TR vs. MT) was used as the within-subject factor, with calcium availability (standard vs. low) and crowding (crowded vs. not crowded) used as the between-subject factors. To assess memory at 3 h (ITM) or 24 h (LTM), training vs. memory test (TR1 vs. TR2 vs. MT) was used as the within-subject factor, and calcium availability (standard vs. low), crowding (crowded vs. not crowded) and the time at which memory was tested (3 h vs. 24 h) were used as the between-subject factors.

Two-way ANOVA was used to analyse the parameters measured in RPeD1 in naïve snails, with calcium condition (standard vs. low) and crowding (crowded vs. not crowded) as factors in the analysis. One-way ANOVA was used to measure the response to training under each of the four stress conditions, with trained condition (naïve vs. 3 h post-training vs. 24 h post-training) as the factor in the analysis. Where significant interactions were found, post-hoc paired t-tests were used to assess within-subject pair-wise differences and Student-Newman-Keuls (SNK) tests were used to assess between-subject pair-wise differences.

### Ethics statement

Ethical approval is not required for research work with *Lymnaea stagnalis*; however every effort was made to ameliorate suffering of animals, ensuring adequate food, clean oxygenated water and low density conditions. The stress treatments used here (outlined above) have no long-term effects on the animals beyond the brief exposure periods. The strain used here is a well established laboratory strain and bred in-house.

## Results

### Electrophysiological activity of RPeD1 in naïve *Lymnaea*

**Figure 1:**
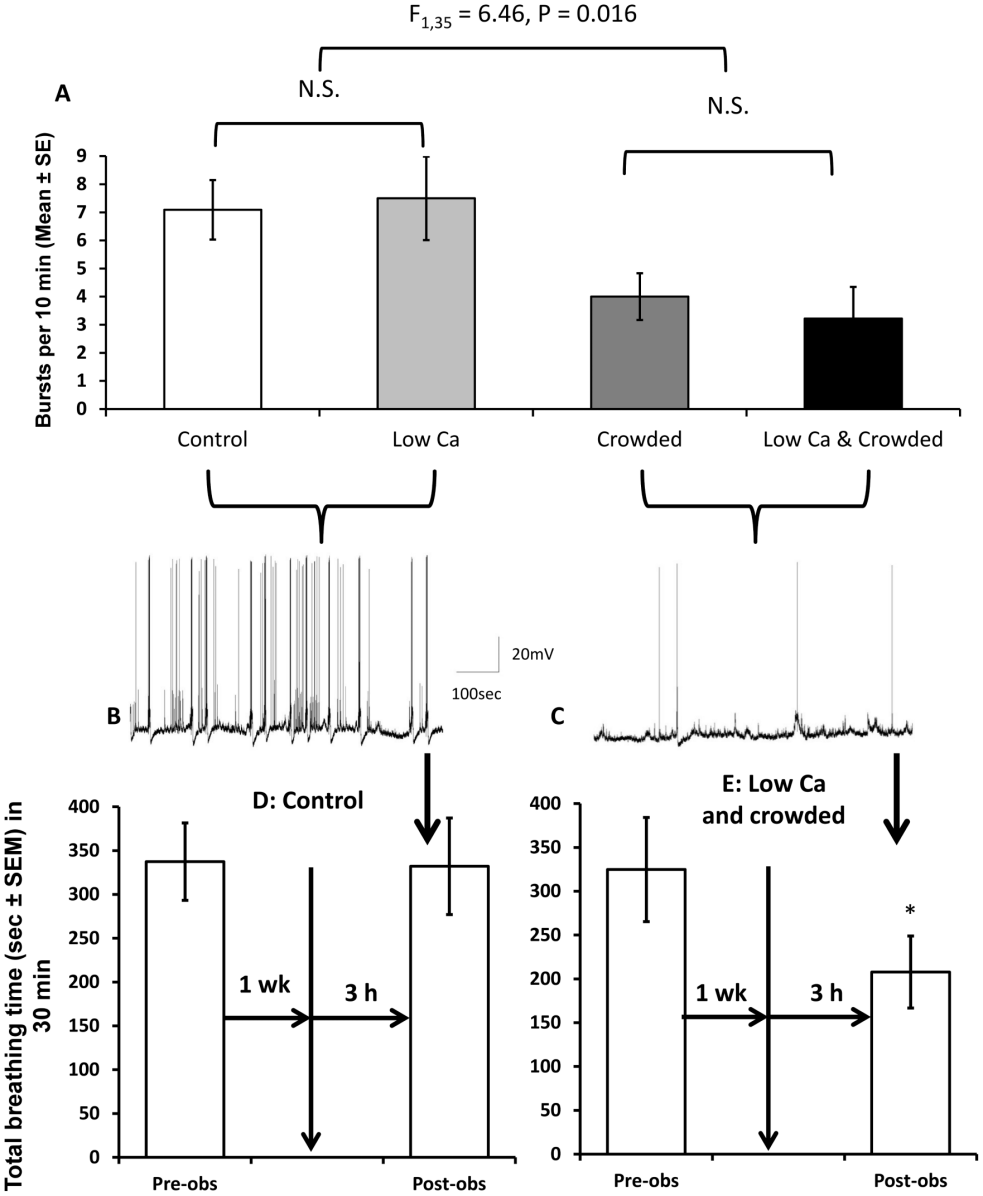
Burst activity in RPeD1 and breathing behaviour 3 h post-stress exposure. RPeD1 burst activity in naïve *Lymnaea*. A) Mean (±SEM) burst activity in untrained *Lymnaea* following exposure to control conditions (white bars: N = 11), low calcium availability (pale grey bars: N = 10), crowding (dark grey bars: N = 9) or a combination of low calcium availability and crowded conditions (black bars: N = 9). Representative traces show burst activity in RPeD1 over 10 min in naïve *Lymnaea* exposed to B) control conditions, which did not differ significantly from those exposed to low calcium only; and C) combined stressors, which did not differ significantly from those exposed to crowding only. Breathing activity in untrained *Lymnaea* 1 week prior to exposure (pre-obs) and 3 h following exposure (post-obs) to D) control conditions (N = 12) or E) combined stressors (N = 12). Control animals did not alter their breathing rate, whereas combined stressors significantly depressed aerial breathing behaviour.

In naïve *Lymnaea*, RPeD1 activity did not differ between snails in control conditions and those exposed to low calcium stress alone (Figure 1) in agreement with previous findings [11]. However, crowding immediately prior to assessing RPeD1 activity altered measured electrophysiological parameters: 1) membrane resistance was significantly increased from 22±2.2 MΩ to 35±4.0 MΩ (ANOVA: main effect of crowding: F_1,34_ = 6.37, P = 0.016); and 2) the number of bursts significantly decreased (Figure 1A: ANOVA: main effect of crowding: F_1,35_ = 6.46, P = 0.016). There was no significant difference in burst activity or membrane resistance between *Lymnaea* experiencing crowded conditions alone versus those experiencing combined stressors, indicating that the responses seen in membrane resistance and bursting activity in naïve snails are due to effects of crowding. The central panel of figure 1 shows examples from: B) bursting activity in an untrained animal held in control conditions throughout; and C) bursting activity in an untrained animal that had been exposed to combined stressors. Excitability did not differ among exposure groups in untrained animals.

### Breathing behaviour

Breathing behaviour did not differ between PreObs and PostObs in animals tested at 10 min following exposure to combined stress or control conditions (PreObs control: 319±50; PostObs control: 344±23; PreObs stressed: 323±37; PostObs stressed: 357±47; N = 12). Combined stress exposure altered TBT during PostObs in animals tested both 3 h and 24 h compared to those exposed to control conditions (rmANOVA: 2-way interaction between time of breathing observation and stress exposure: F_1,42_ = 4.65, P = 0.037). At 3 h post-exposure there was no difference between PreObs and PostObs in animals exposed to control conditions throughout (Figure 1D: Paired t-test: t = 0.16, P = 0.874, N = 12); however, following exposure to combined stressors total breathing time in naïve snails was significantly reduced (Figure 1E: Paired t-test: t = 2.21, P = 0.049, N = 12). Similarly, 24 h post exposure control animals demonstrated no difference between PreObs and PostObs (PreObs: 340±55; PostObs: Paired t-test: 315±52; t = 0.30, P = 0.768, N = 11); however animals exposed to stress significantly decreased their TBT between PreObs and PostObs (PreObs: 338±60; PostObs: 178±42; Paired t-test: t = 2.91, P = 0.016, N = 11).

### Yoked controls

**Figure 2:**
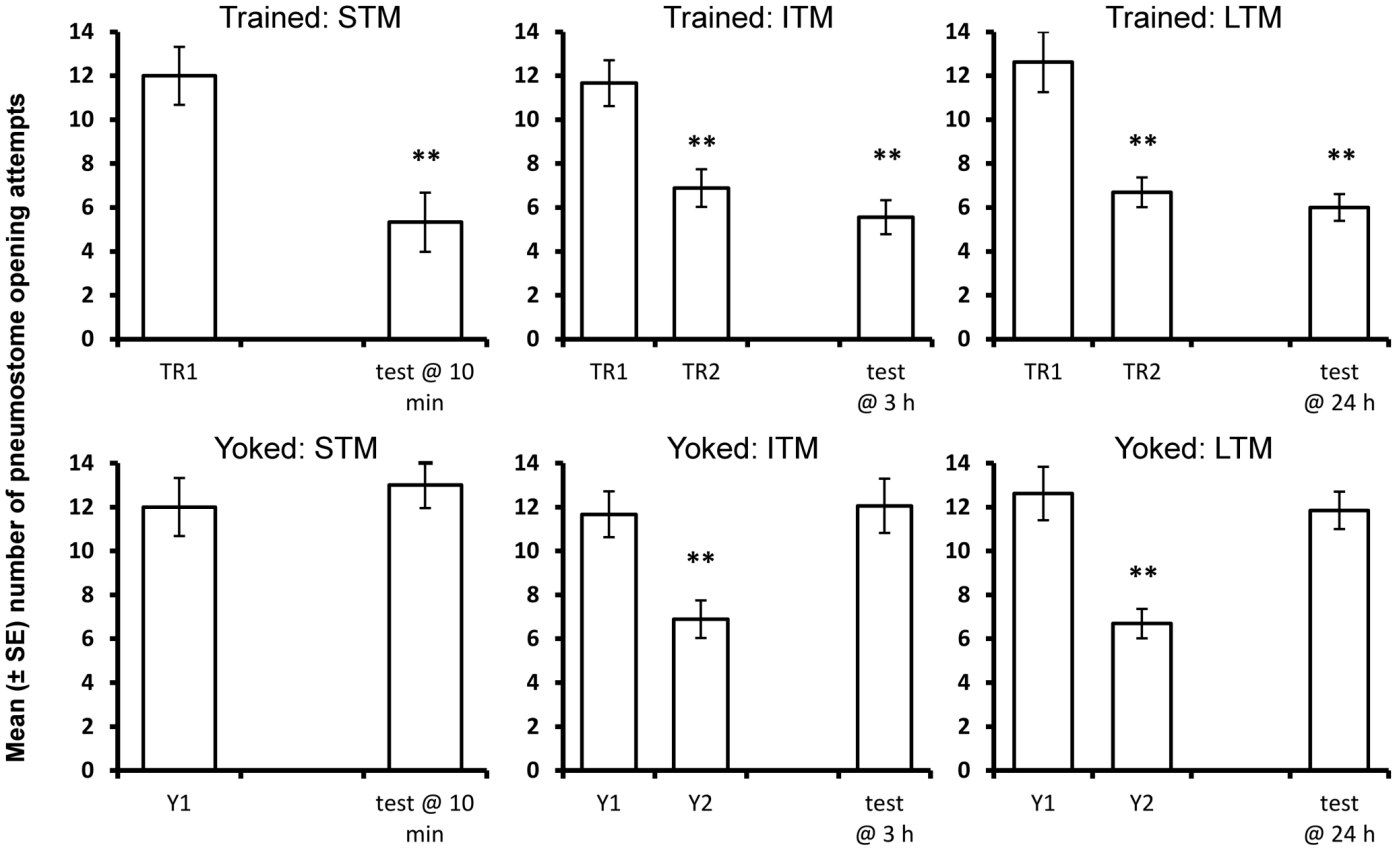
Comparison between operantly trained and yoked training procedures in control conditions. *Lymnaea* demonstrated memory at all three time periods following operant conditioning, but breathing attempts during the memory test did not differ significantly from the number of stimuli during Y1 following yoked-control training (STM: N = 12; ITM: N = 18; LTM: N = 13). **  =  significantly different from Y1 or TR1 (P<0.01, paired t-test).

*Lymnaea* showed a significant reduction in pneumostome opening attempts following operant conditioning in control conditions 10 min following training (STM), but did not demonstrate a reduction in breathing attempts in yoked animals (Figure 2: rmANOVA: 2-way interaction between training protocol and response to training: F_1,22_ = 7.10, P = 0.014). Similarly, *Lymnaea* demonstrated a reduction in breathing attempts at both 3 h and 24 h during the memory test following operant conditioning, but did not demonstrate a reduction in breathing attempts in yoked animals (Figure 2: rmANOVA: 2-way interaction between response to training and type of training received: F_2,116_ = 12.50, P<0.001). Therefore, the reduction in aerial respiration following operant conditioning is not a generalised response to exposure to hypoxia or a tactile stimulus, but is dependent on memory following a contingent stimulus associated with an attempt to breathe.

### Behavioural assessment following operant conditioning

**Figure 3:**
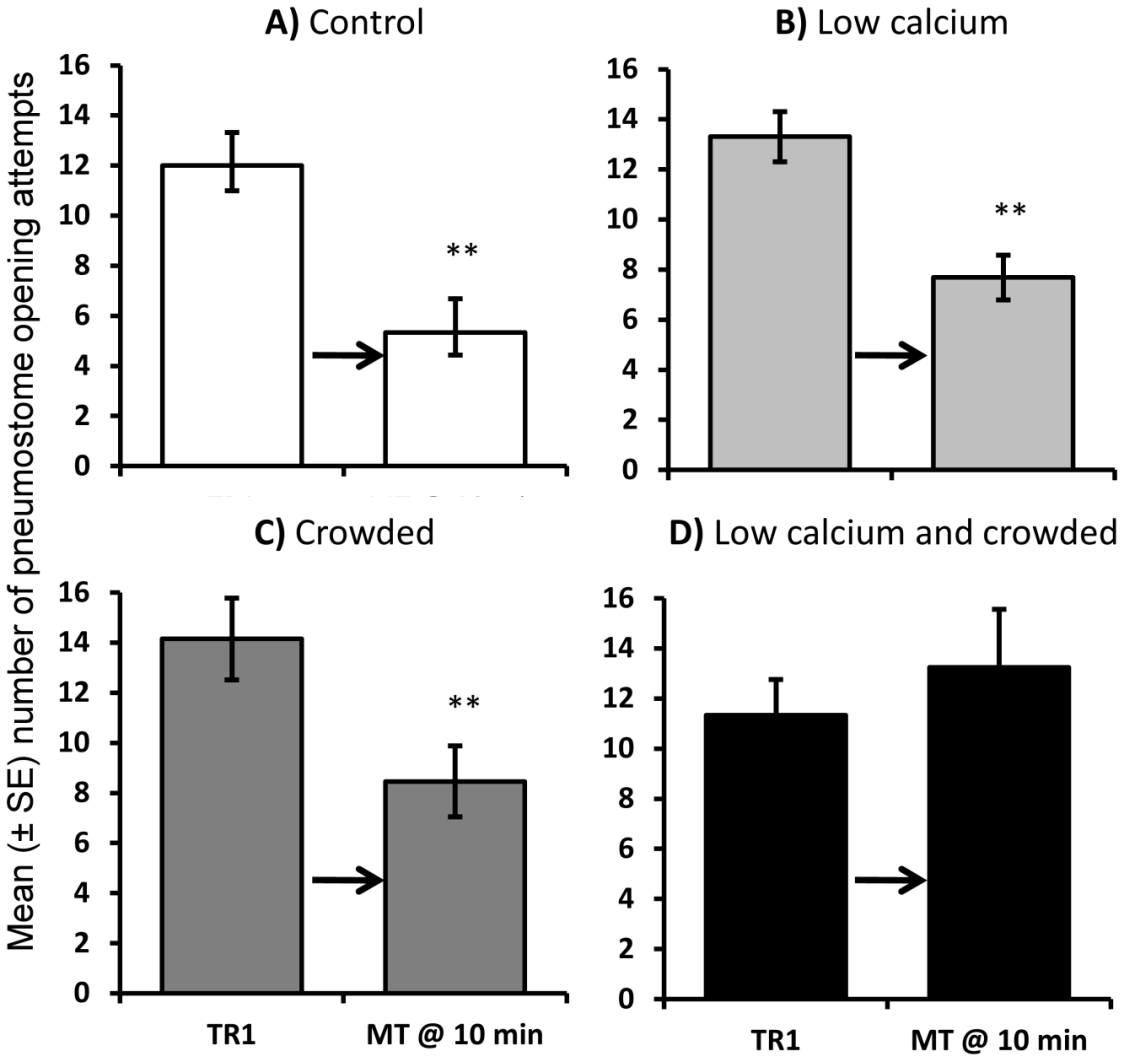
Behavioural assessment of short-term memory (STM). Mean (± SEM) number of pneumostome opening attempts during training (TR1) and the test for short-term memory 10 min following training (MT @ 10 min). *Lymnaea* were exposed to: A) control conditions (white bars; N = 12); B) low calcium availability (pale grey bars; N = 13); C) crowding (dark grey bars; N = 13) or D) a combination of low calcium availability and crowded conditions (black bars; N = 12). **  =  significant difference between training and the memory test (P<0.01, paired t-test).

Following a single training session stress exposure altered whether *Lymnaea* form short-term memory (STM: 10 min). In control conditions and following exposure to either stressor alone *Lymnaea* exhibited a significant depression in breathing attempts during the memory test demonstrating STM formation; however, following exposure to combined stressors *Lymnaea* did not reduce the number of breaths during the memory test indicating that STM had been blocked (Figure 3: rmANOVA: 3-way interaction between response to training, calcium level and exposure to crowding: F_1,46_ = 4.10, P = 0.049). The initial number of breathing attempts during training did not differ significantly among treatment groups (SNK: P > 0.05 for all pair-wise comparisons).

**Figure 4:**
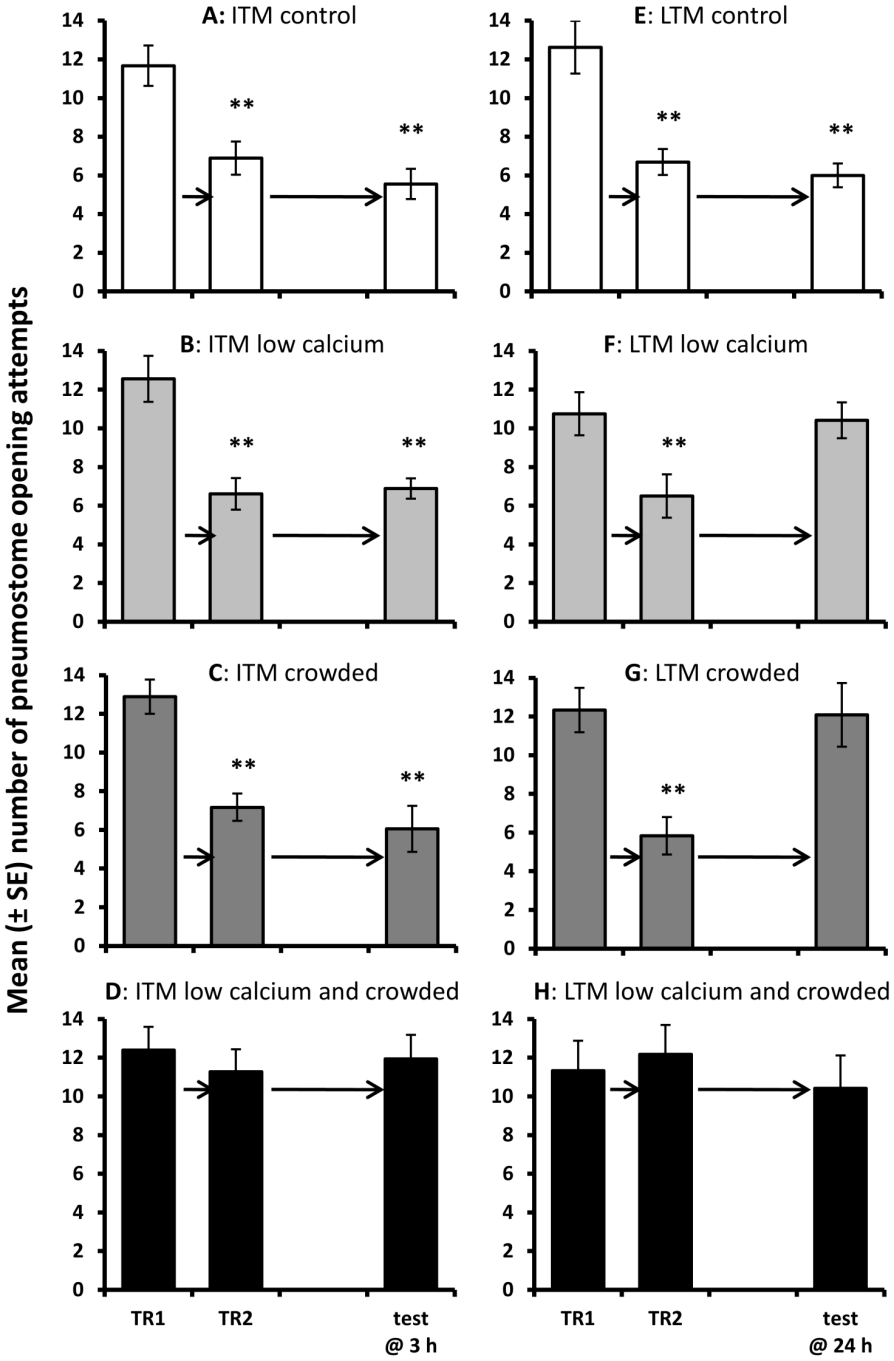
Behavioural assessment of intermediate-term and long-term memory. Mean (± SEM) number of pneumostome opening attempts during training (TR1 and TR2) and the test for intermediate-term memory 3 h following training (A to D: test @ 3 h) or long-term memory 24 h following training (E to H: test @ 24 h). *Lymnaea* were exposed to:control conditions (white bars: A: N = 18; E: N = 13); low calcium availability (pale grey bars: B: N = 18; F: N = 12); crowding (dark grey bars: C: N = 18; G: N = 12) or a combination of low calcium availability and crowded conditions (black bars: D: N = 18; H: N = 12). **  =  significant difference between the first training session (TR1) and second training session (TR2) or the memory test (P<0.01, paired t-test).

Memory formation following two half hour training sessions separated by a 1 h interval depended on both stress exposure and the time at which memory was tested (Figure 4: 4-way interaction between response to training, calcium level, exposure to crowding and timing of MT: F_2,226_ = 7.13, P = 0.001). In control conditions *Lymnaea* demonstrated both ITM and LTM formation (Figure 4A: TR1 vs. MT 3 h: t = 6.63, P<0.001; Figure 4E: TR1 vs. MT 24 h: t = 4.26, P = 0.001). Following exposure to either stressor experienced individually *Lymnaea* demonstrated ITM 3 h following training (Figure 4B: low calcium only TR1 vs. MT 3 h: t = 4.51, P<0.001; Figure 4C: crowding only TR1 vs. MT 3 h: t = 4.76, P<0.001) but not LTM 24 h following training (Figure 4F: low calcium only TR1 vs. MT 24 h: t = 0.24, P = 0.813; Figure 4G: crowding only TR1 vs. MT 24 h: t = 0.27, P = 0.795). However, following exposure to both stressors combined *Lymnaea* failed to demonstrate either ITM or LTM (Figure 4D: TR1 vs. MT 3 h: t = 0.23, P = 0.818; Figure 4H: TR1 vs. MT 24 h: t = 0.37, P = 0.721). The difference in memory formation between the stress exposure groups was not due to differences in pneumostome opening attempts during the first training session as initial number of breathing attempts during TR1 did not differ among treatment groups (SNK: P > 0.05 for all pair-wise tests). However, the number of pneumostome opening attempts during TR2 was significantly greater in the combined stress groups compared to both control and single stress groups (SNK: P<0.05 for each pair-wise comparison), indicating that *Lymnaea* did not demonstrate learning when exposed to combined stressors.

Together these data demonstrate that in control conditions *Lymnaea* forms STM, ITM and LTM. Following exposure to either low calcium or crowding alone *Lymnaea* forms STM and ITM, but LTM formation is blocked, in agreement with previous findings [11], [16]–[18]. However, following exposure to a combination of low calcium availability and crowded conditions all memory processes (STM, ITM and LTM) are blocked when assessed behaviourally.

### Electrophysiological activity in RPeD1 following training

**Figure 5:**
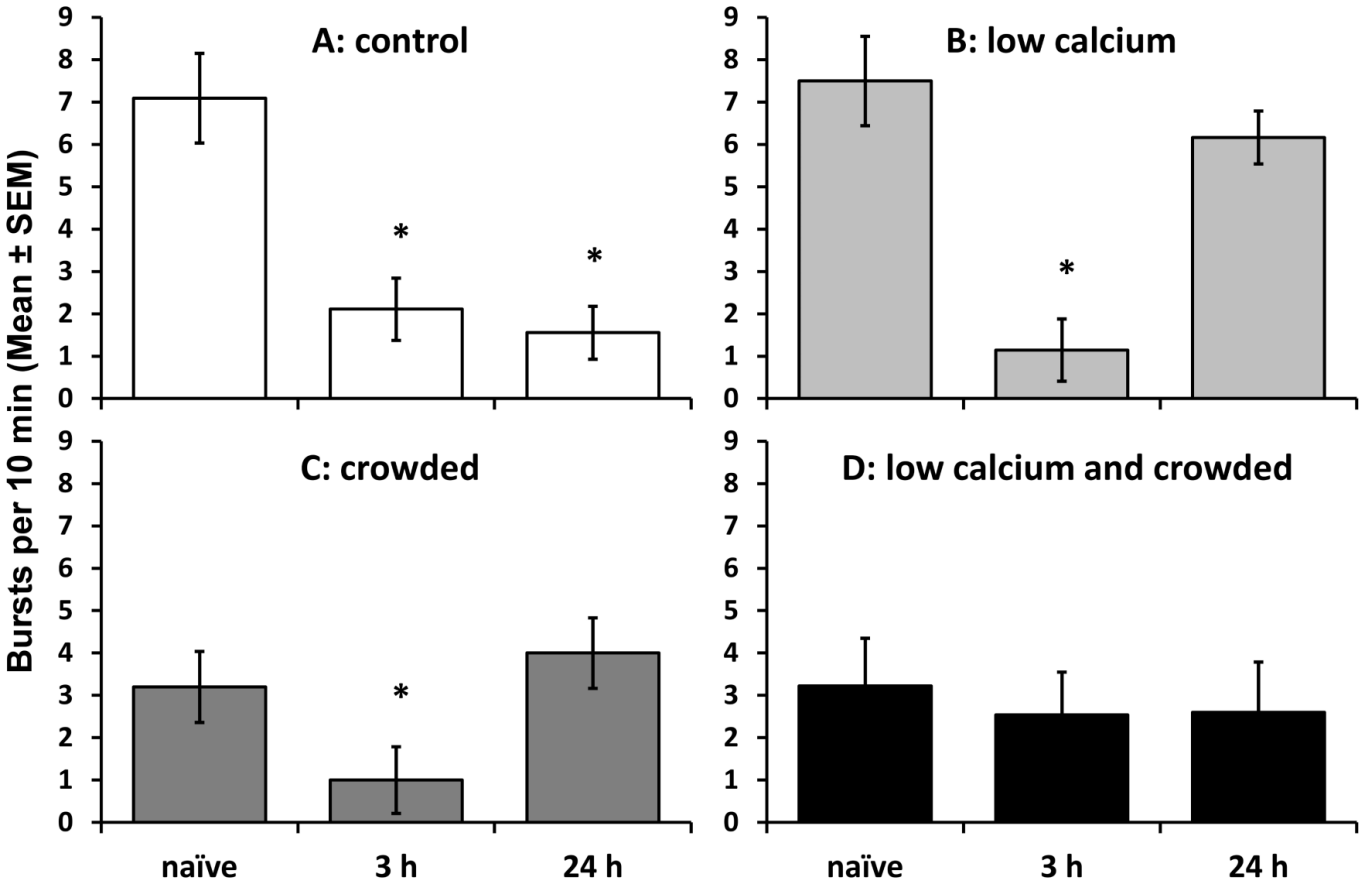
RPeD1 burst activity in naïve versus trained *Lymnaea*. Mean (±SEM) burst activity in RPeD1 in naïve *Lymnaea*, 3 h following training (i.e. representing intermediate-term memory test) and 24 h following training (i.e. representing long-term memory test) following exposure to: A) control conditions (white bars: naïve: N = 11; ITM: N = 9; LTM: N = 9); B) low calcium availability (pale grey bars: naïve: N = 10; ITM: N = 7; LTM: N = 6); C) crowding (dark grey bars: naïve: N = 9; ITM: N = 10; LTM: N = 10) or D) a combination of low calcium availability and crowded conditions (black bars: naïve: N = 9; ITM: N = 13; LTM: N = 10). *  =  significant difference between naïve and trained bursting behaviour (P<0.05: SNK test).

In control conditions, burst activity in RPeD1 is significantly decreased both 3 h and 24 h following training relative to the naïve, untrained state (Figure 5A: ANOVA: F_2,26_ = 13.01, P<0.001; naïve vs. 3 h, SNK: P<0.05; naïve vs. 24 h, SNK: P<0.05). Following exposure to the low calcium stress alone, bursting was significantly depressed 3 h following training (Figure 5B; naïve vs. 3 h, SNK: P<0.05) but not 24 h later (Figure 5B; ANOVA: F_2,26_ = 4.30, P = 0.028; naïve vs. 24 h, SNK: P > 0.05). Similarly, despite starting at an already depressed level in the naïve, untrained state, burst rate decreased further at 3 h following training in snails that were crowded only (Figure 5C; naïve vs. 3 h, SNK: P<0.05), but did not differ significantly from naïve animals 24 h following training (Figure 5C: ANOVA: F_2,26_ = 3.57, P = 0.043; naïve vs. 24 h, SNK: P > 0.05). When *Lymnaea* were exposed to the combined stressors there was no significant change seen in burst activity in RPeD1 following training relative to the naïve state (Figure 5D; ANOVA: F_2,29_ = 0.11, P = 0.898). Membrane resistance and excitability showed no significant change following training relative to the naïve state in any of the stress exposure groups.

Therefore, the change in burst activity of RPeD1 following training correlates well with behavioural responses to training in each of the stress conditions. RPeD1 demonstrates a significant decrease in burst rate relative to the naïve state, irrespective of whether the naïve state is already depressed, at times equivalent to when we find behavioural reduction in pneumostome opening attempts during MT.

## Discussion

Stress is considered any condition that seriously perturbs the physiological or psychological homeostasis of an organism [28]. One common consequence of stress exposure is the alteration of the ability to form memory [1], [2], [6]. The majority of studies to date designed to understand the mechanism by which this occurs assess the effects of individual stressors (but see work by Zoladz et al.) [29], [30]; however, in ‘real life’ different sources of stress may be experienced concurrently. Here we demonstrate that different forms of stress (crowding and low environmental calcium availability) which produce a similar behavioural phenotype, i.e. blocking LTM but not ITM or STM, differ in the way they affect activity of a neuron (RPeD1), known to be necessary for LTM formation [9], [25]. When experienced together these stressors had an additive effect blocking all memory processes.

Exposure to either a low calcium environment or crowding immediately prior to training blocked the ability of *Lymnaea* to form LTM but not ITM in agreement with our previous findings [11], [16], [17]. At the neuronal level burst activity in RPeD1 has been found to be necessary in driving pneumostome opening during aerial respiration. Bursts in RPeD1 in naïve *Lymnaea* did not differ from controls in response to low environmental calcium [11]. However, following crowding naïve untrained *Lymnaea* showed a significant reduction in the number of bursts recorded in RPeD1. Thus, despite having apparently identical effects on behavioural phenotype in the Dutch *Lymnaea*, the two stressors affect RPeD1, the neuron that initiates CPG rhythmogenesis and is necessary for LTM formation [9], [25], in a significantly different manner. This finding is consistent with earlier findings, that the sensory pathways mediating the two stressors were different [18], and their effect on memory formation in other strains of *Lymnaea* differs [19], both of which indicated the way these stressors are processed in the CNS may not be the same.

It has been suggested [4] that the mammalian CNS parses stressors into two categories: ‘physical’ (e.g. loss of blood) or ‘psychological’ (e.g. restraint). Physical and psychological stressors differ in their effects on certain neuronal plasticity-related genes (e.g. Gap-43, CREB) in neurones of different brain areas; as well producing temporal differences in plasma corticosterone levels [31]. In addition, it had been shown that different dopamine receptors (D_1_ and D_2_) mediated the neuronal response to two different stressors in the medial pre-frontal cortex (mPFC) [32]. It was suggested that the role of the dopamine receptors in the mPFC differs depending upon whether the stressor is a physical or a psychological one, and the modulation of subcortical brain regions and the hypothalamic-pituitary-adrenal (HPA) axis is thus differentially affected. Thus ‘strong’ physical stressors appear to activate both mPFC D_1_ and D_2_ receptors while ‘milder’ psychological stressors activate only mPFC D_2_ receptors. RPeD1 has been identified as a dopaminergic neuron [33]; however the nature of the receptors in this neuron have yet to be determined so we are unable to judge at this time whether receptor activity differs dependant on the type of stress experienced. Additionally, whether we can parse the two stressors here into such a categorisation is uncertain, but worth consideration. We suggest that crowding may be a ‘psychological’ stressor whereas the low calcium environment is a ‘physical’ stressor. These two stressors presented independently produce the same behavioural phenotype (i.e. block LTM), yet have significantly differing effects on plasticity in the CNS. In RPeD1, the low calcium environment did not alter input resistance, the resting membrane potential (RMP) neuronal excitability (i.e. the number of action potentials elicited by a depolarizing pulse) or the number of bursts in RPeD1 in naïve *Lymnaea*. However, crowding significantly decreased the number of RPeD1 bursts while significantly increasing RPeD1’s input resistance in naïve animals.

At first glance the obtained electrophysiological data following stress exposure in naïve *Lymnaea* seem inconsistent. While it has been straightforward to show for example that certain individual stressors such as predator kairomones and KCl alter behaviour and the activity of RPeD1 in a predicable manner [12], the same does not hold true for all stressors or when individual stressors are combined as demonstrated here. This is due to the complexity of the sensory systems mediating each of the stressors and the fact that the intrinsic membrane property (e.g. membrane resistance) is not the only important variable that alters aerial respiratory behaviour. RPeD1’s activity in the 3-neuron CPG that drives aerial respiratory behaviour is the result of emergent network properties due to complex interactions between the 3 neurons [8], [34]. The intrinsic membrane properties of each of the neurons as well as the synaptic interactions between them will have to be examined in order to come to an understanding of how stressors when experienced together alter aerial respiration. Thus, it is not straightforward to ascribe a change in the membrane input resistance of RPeD1 with bursting activity.. In the low calcium environment we see a change in the way RPeD1 responds to training without any effect on naïve animals. With crowding, on the other hand, the input resistance increased yet the number of bursts significantly decreased. This does not appear to make sense, since a higher input resistance should mean that any synaptic input coming onto RPeD1 should be larger and thus if it were an excitatory input it should result in increased activity, not a decrease. However, since bursting requires a precise interplay of activity between all three members of the CPG (the other two being VD4 and IP3I) it is probable that a change in input resistance in RPeD1 could upset this balance. Until we record from the three CPG neurons simultaneously (which is extremely difficult if impossible to do) we are unable to say with any certainty why there is a significant decrease in the number of bursts produced in RPeD1 as a result of crowding.

We have previously ascribed declines in aerial breathing behaviour following operant conditioning to corresponding declines in bursting in RPeD1 [11], [23], [26]. However, it has been shown that following LTM formation, causing RPeD1 to burst by the injection of depolarizing current does not in the vast majority of cases cause a pneumostome opening [10]. Whether a burst results in pneumostome opening also appears to change in response to stress exposure. For example, we found declines in aerial breathing rate in a low calcium environment [21] whilst RPeD1 burst behaviour remains unchanged in untrained animals [11]. Similarly, in crowded snails aerial breathing behavior declines in freely breathing individuals [16], but in this case RPeD1 burst activity also decreases in untrained animals. We also see a significant reduction in RPeD1 bursts in response to joint stressors compared to control conditions in naïve *Lymnaea* following stress exposure, which again correlated with a decline in total breathing time in combined stress conditions. Therefore, the two stressors used here differ in their effect on RPeD1 burst activity, but not their effect on aerial respiration. Interestingly, we see no such decline in total breathing time 10 min following stress exposure, which may be due to an increased drive to breathe as the animals do not have a chance to recover from the hypoxic environment. Unfortunately we were unable to assess RPeD1 activity at this time point due to the time dissection and recovery takes, so are unable to show whether burst activity remains unaltered at this time.

Whilst we see significant changes in TBT and RPeD1 burst activity 3 h and 24 h following combined stress exposure, there is no concurrent decline in pneumostome opening attempts during the first training session. This demonstrates firstly that aerial breathing behaviour in freely breathing *Lymnaea* is not directly related to burst activity in RPeD1 as we also see a decline in TBT in animals exposed only to low calcium conditions only [21]. Secondly, it shows that the number of pneumostome opening attempts during training in the presence of a physical stimulus is also not directly related to breathing behaviour in the absence of physical stimuli. Therefore the relationship between burst activity in RPeD1, breathing behaviour and breathing attempts during training is not a direct relationship, and appears to be modulated by environmental stressors in naïve *Lymnaea*. Importantly, the lack of change (10 min) or depression (3 h and 24 h) in TBT also indicates that the lack of memory following exposure to combined stressors was not due to an increase in breathing behaviour masking memory during these time points.

In the low calcium environment operant conditioning results in ITM (3h following training) but not LTM. The same behavioural phenotype is seen with crowding. In fact, at the behavioural level the data for *Lymnaea* experiencing crowded or low calcium conditions do not differ significantly in either their effect on total breathing time as found previously [16], [21] or during testing for ITM as seen both on previous occasions and again here [11], [16], [18]. Similarly, 3 h following training (i.e. ITM) *Lymnaea* exposed to either crowded or low calcium conditions alone show a significant decrease in the number of bursts in RPeD1. This occurs in crowded conditions despite the number of bursts in naïve animals already being in a significantly depressed state. Therefore, an initial reduction in burst behaviour in RPeD1 did not prevent further plasticity following training. These data confirm our previous findings, that where we show behavioural memory is formed, burst behaviour in RPeD1 is also significantly decreased relative to the naïve state [11], [23], [26], showing that the state in RPeD1 correlates well with the state of the animal (i.e. trained vs. untrained). However, it adds a caveat to this finding, that we must first know the state of the naïve animal prior to training, as a direct comparison between RPeD1 in a control and a crowded untrained snail would indicate (falsely) that the crowded animal has formed memory.

Previous work has shown that combining stressors with differing effects on phenotype may not alter memory formation in a predictable manner [5]. For example, by combining low calcium (as used here) with social isolation, which alone had no effect on memory, low calcium no-longer had a blocking effect on memory [5]. This was thought due to a reduction in calcium requirements in isolated animals as their drive to mate in the male role is increased [35], potentially decreasing the calcium they require for egg-laying [36]. However, here stressors with a similar effect on phenotype (blocking LTM) appear to have an additive effect, i.e. blocking all memory processes, which may be due to the animal simply experiencing too much stress to pay attention to training. This was thought to be the reason why combining three different potential stressors, isolation, low calcium and predator kairomones, blocked the ability of *Lymnaea* to form LTM [5]. Alternatively, it may be specific to the type of stress experienced. For example, in crowded conditions *Lymnaea* may be experiencing competition for limited calcium resources. Further work is required to assess how both the individual action of a stressor and the biological relevance to the species may play into predicting how a particular stressor will act in combination with other forms of stress in altering memory formation.

Together the data presented here raise three important points. Firstly, that the effect different stressors have may produce the same behavioural phenotype, but have different effects within the CNS (i.e. activity in RPeD1). Secondly, to assess whether electrophysiological activity in the CNS of an animal reflects its current state (i.e. whether memory has been formed), we must first know the conditions experienced by the naïve animal and how this alters CNS activity in untrained animals. Thirdly, stressors experienced in combination can have additive effects on the ability of an animal to form memory, and may block all memory processes.
